# Small Islets Transplantation Superiority to Large Ones: Implications from Islet Microcirculation and Revascularization

**DOI:** 10.1155/2014/192093

**Published:** 2014-04-16

**Authors:** Wenjuan Li, Ruxing Zhao, Jidong Liu, Meng Tian, Yiran Lu, Tianyi He, Meng Cheng, Kai Liang, Xia Li, Xiangdong Wang, Yu Sun, Li Chen

**Affiliations:** ^1^Department of Endocrinology, Qilu Hospital of Shandong University, Institute of Endocrinology and Metabolism, No. 107 West Wenhua Road, Jinan, Shandong 250012, China; ^2^Department of Poisoning and Occupational Disease, Qilu Hospital of Shandong University, Jinan 250012, China; ^3^Institute of Cell Biology, Shandong University School of Medicine, Jinan 250012, China

## Abstract

Pancreatic islet transplantation is a promising therapy to regain glycemic control in diabetic patients. The selection of ideal grafts is the basis to guarantee short-term effectivity and longevity of the transplanted islets. Contradictory to the traditional notion, recent findings implied the superiority of small islets for better transplantation outcomes rather than the large and intact ones. However, the mechanisms remain to be elucidated. Recent evidences emphasized the major impact of microcirculation on islet **β**-cell mass and function. And potentials in islet graft revascularization are crucial for their survival and preserved function in the recipient. In this study, we verified the distinct histological phenotype and functionality of small islets versus large ones both in vitro and in vivo. With efforts to exploring the differences in microcirculation and revascularization of islet grafts, we further evaluated local expressions of angiotensin and vascular endothelial growth factor A (VEGF-A) at different levels. Our findings reveal that, apart from the higher density of insulin-producing **β**-cells, small islets express less angiotensin and more angiotrophic VEGF-A. We therefore hypothesized a logical explanation of the small islet superiority for transplantation outcome from the aspects of facilitated microcirculation and revascularization intrinsically in small islets.

## 1. Introduction

Diabetes mellitus is now ranking among the top list of diseases leading to mortality and disability in human [1]. Type 1 diabetes mellitus is characterized by the autoimmune destruction of the pancreatic insulin-producing *β*-cells and subsequently absolute deficiency of insulin to maintain glucose homeostasis [2]. In later stages of type 2 diabetes mellitus, the failure of islet *β*-cells often plays a core role in the decompensated glucose homeostasis [3]. Therefore, most diabetic patient, regardless of the current classification, would be faced with the indispensable replacement therapy of insulin and eventually become “insulin dependent” [4]. Currently, exogenous insulin injection generally serves as the most effective regime [4]. However, administration of insulin is onerous for the patients. And most importantly, due to the breach of the closed dynamic regulation loop in response to physiological changes, it is difficult for exogenous insulin formulations to avoid episodes of hyperglycemia and hypoglycemia [5]. As first introduced by the Edmonton protocol in the year of 2000, pancreatic islet transplantation (PIT) emerged as a promising method to normalize metabolic control in a way that cannot be achieved with exogenous insulin [6–8]. Great successes have been achieved with this treatment plan [8–11]. However, there are still several hurdles before the real success [12]. The first major hurdle is the source and choice of more qualified grafts which directly affect the short-term effectivity and long-term survival of the transplanted islets [4].

It has been traditionally regarded that large and intact islets during isolation were of better choice for transplantation. However, in the past decade, evidences have emerged indicating that small islets, both in vitro and in vivo, have more insulin content [13] and stronger secretory function [14, 15]. Better outcomes were also observed in small islets transplantation than in large ones [15]. However, the mechanisms underlying the superiority of small islets in transplantation remain largely to be elucidated [16].

A series of work has been done regarding the distinctive phenotypes between small and large islets [8, 17]. Previous studies mainly focused on the phenotypic and function divergence in insulin-producing *β*-cells or other endocrine cells. However, as one of the most vascularized organs, pancreatic islets consist of a network of specialized capillaries that regulate islet blood flow as well as endocrine cells function [18–20]. To note, in PIT, the islet microcirculation and revascularization were of determinant importance in both functionality and longevity of transplanted grafts [20–24]. Therefore, the divergence in microcirculation and revascularization would have causative role in distinct function and outcomes between different groups of islets.

In this study, we verified the distinct histological phenotype and functionality of small islets versus large ones both in vitro and in vivo. We explored the divergence in microcirculation as well as expressions of local dominant regulators of microcirculation and revascularization such as angiotensin and VEGF-A.

## 2. Material and Method

### 2.1. Rat Islet Isolation

Rat islet isolation was performed according to the pancreas in situ perfusion method previously described with minimal modification. Generally, primary rat islets were derived from health adult Wistar rats with body weight (250 g), age (10~12 weeks), and sex (male) matched. After sterilization, the operation was performed in sodium pentobarbital anesthetized rats. Common bile duct was exposed, then punctured using syringe with 28 G needle, and injected with 6 mL cold 1 mg/mL collagenase V (Sigma, catalog number: C9263). The engorged pancreas was removed and incubated in Hanks solution for 12 min at 37°C and then shook briefly into sand-like mixture. The sand-like mixture was infiltrated by 50-mesh sieve and washed with Hanks solution. The pellet was resuspended with 5 mL Histopaque 1077 (Sigma, catalog number: 10771). 10 mL Histopaque 1077 was subsequently added to the mixture slowly. And the preparation above was filled with 10 mL Hanks solution carefully to keep the interface intact and then centrifuged for 15 min at 800 g without brake. Finally, we collected islets in the interface between Hanks solution and Histopaque 1077 using 1 ml pipette and washed 3 times with Hanks solution for further experiment.

### 2.2. Islet Size Determination and Sorting of Small versus Large Islets

Triplicate samples of each batch of islets, each comprising about 5% of the total islet fraction from a single pancreas, were transferred into 24-well plate and examined under light microcopy. The diameter of the individual islet was determined and recorded for calculating total islet volume. For irregularly shaped islets, diameter measurements by two vertical axes on the islet were taken and the average was used. Islet volumes were calculated and converted to islet equivalents for the sample and the entire islet fraction. Diameters were calculated by islet equivalent (IEQ, islet of diameter every 150 *μ*m is defined as 1IEQ).

The rat islets were sorted into 2 groups manually using blunted syringe. Based on previous reports concerning large and small rat islets, small islets were defined as islets with a diameter of less than 125 *μ*m, whereas large ones with a diameter of over 150 *μ*m. Islets between 125 and 150 *μ*m diameters were excluded from analysis, in order to obtain clean group classification.

### 2.3. Diabetic Models Establishment and Pancreatic Islets Transplantation

Age matched syngeneic rats (males, 280–300 g) diabetic models were generated by intraperitoneal injection of streptozotocin (STZ, 85 mg/kg body weight; Sigma) freshly dissolved in citrate buffer (pH 4.5). One week after the successful diabetic modeling confirmed by three days of glucose monitor (random glucose levels >16 mmol/L), diabetic rats were randomly distributed into 3 groups (for each *n* = 6): small islets (as defined above) transplantation group, large islets transplantation group, and sham-operation group. Twenty-four hours after isolation, 3,000 IEQ were injected into the portal vein via 25 G needle connected to syringe as previously described [9].

### 2.4. Intraperitoneal Glucose Tolerance Test

Before and after the successful induction of diabetes, blood glucose levels were monitored by tail vein puncture with an Accu-chek glucometer (Roche, Mannheim, Germany). The transplantation outcomes were evaluated by routine intraperitoneal glucose tolerance test (IPGTT) at the 10th day and 40th day after PIT operation. Generally, after overnight-fasting, rats were injected intraperitoneally with 2 g/kg glucose. Blood glucose levels were obtained at indicated time points (0, 30, 60, 90, 120, and 180 min). IPGTT curves were drawn and areas under curve (AUCs) were also calculated for transplantation outcomes evaluation using Graphpad Prism 5.01.

### 2.5. In Vitro Islet Culture and Insulin Secretion Assay

Rat islets were cultured at 37°C with 5% CO_2_ in RPMI 1640 medium (Gibco) supplemented with 10% FBS, 25 mmol/L HEPES (Gibco), 50 U/mL of penicillin, and 50 ug/mL of streptomycin. 30–50 islets were put into each well of the 6-well plate. During culture, shake the plate to avoid gathering. In accordance with the routine culture, fresh medium is replaced with half of the total amount.

To evaluate and compare the secretion function of small and large islets graft in vitro, glucose stimulated insulin secretion assay was performed. Islets of total 20 normalized IEQs were transferred into each well of 24-well plate with 1.5 mL serum-free RPMI 1640 medium. After overnight incubation at 37°C with 5% CO_2_, islets were washed 3 times with glucose-free medium and then incubated in fresh medium containing 2.8 mM glucose or 16.7 mM glucose for 2 hours. The supernatant was collected for insulin level assay. The insulin levels in the medium were determined by radioactive immunoassay in Institute of Experimental Nuclear Medicine, Shandong University. Triplicate samples were tested for each condition. And three independent experiments were arranged in our study.

### 2.6. Islet Viability Assay

Cultured islets were examined under light microscopy and recorded at indicated time points (24 h, 72 h, and 120 h). Isolated islets showed a concentrated necrosis pattern with prolonged culture in vitro. This was mainly presented as a reduction in light transmittance morphologically.

To further study difference of the islet grafts viability between large and small islets, we quantified the islet cells viability by a colorimeter-based assay. Single islet cell was obtained immediately after islet isolation. Freshly isolated islets were digested at 37°C for 17 min with 0.25% EDTA-trypsin (Sigma) followed by syringe injection through progressively narrower needles sized from 16 to 22 G to disperse into single-cell suspension [5]. The viability of islet cells seeded in a 96-well plate (5000 cells/well) was immediately determined using CCK-8 kit (Beyotime, CN). The WST-8 [2-(2-methoxy-4-nitrophenyl)-3-(4-nitrophenyl)-5-(2,4-disulfophenyl)-2H-tetrazolium, monosodium salt] in the CCK-8 kit produced a water-soluble formazan dye upon reduction in the presence of an electron carrier in viable cells. The light absorbance of the medium was measured at 450 nm using microplate reader. All samples were determined in triplicate.

### 2.7. Tissue Total RNA Extraction and Quantitative Reverse-Transcription PCR

Total RNA was extracted and purified from isolated fresh islets homogenized using TRIzol Reagent (Invitrogen, CA, USA) (purity >1.75) and synthesized at once into cDNA using a RevertAid First Strand cDNA Synthesis Kit (Thermo Scientific) according to the manufacturer's manual. Briefly 2 ug of total RNA was reverse transcribed in a system containing 5x buffer reverse transcriptase, Rnase inhibitor, poly T primers, 100 mM dNTP Mix, and a total volume of 20 uL. The reaction was carried out in iCycler thermocycler (Bio-rad, Germany) at 42°C for 1 h and 70°C for 5 min. The cDNA products were stored at −80°C for further quantification assay.

Real time quantitative PCR for rat indicated genes was performed in the LightCycler 480 System (Roche Applied Science) using SYBR green (Toyobo) as a dye reagent with 40-cycle protocol. After initial denaturation (95°C, 5 minutes), PCR reactions were conducted with the following parameters: denaturation at 95°C for 15 seconds, annealing at 65°C for 15 seconds, and extension at 72°C for 45 seconds. The primer sequences used for amplification of rat genes were as follows:

Rat* VEGF-A*: sense, 5′-TGCCAAGTGGTCCCAG-3′; and antisense, 5′-CGCACACCGCATTAGG-3′;

rat* Angiotensinogen*: sense, 5′-TTCAGGCCAAGACCTCCC-3′; antisense, 5′CCAGCCGGGAGGTGCAGT-3′;

rat* AT1*: sense, 5′-TTCAGCCAGTGTTTTAGA-3′; antisense, 5′-TTACTCCTTGGA-GGCCATGT-3′ [2];

rat* GAPDH*: sense, 5′-ACTCCCATTCCTCCACCTTT-3′; and antisense, 5′-TTACTCCTTGGA-GGCCATGT-3′. The amplification efficiency of the PCR products was calculated according to the Ct values. Target gene expressions were demonstrated relative to the number of GAPDH transcripts used as the internal reference. All samples were detected in triplicate.

### 2.8. Western Blotting

Extraction of whole tissue protein was conducted with RIPA (strong). Protein was quantified with BCA protein assay kit. 20 ug of protein was separated by SDS-PAGE. After electrophoresis, protein was transferred to PVDF membranes which was blocked with 5% nonfat milk in TBS-T (10 mM Tris, 150 mM NaCl, and 0.05% Tween-20) for 2 h. And then PVDF membrane was washed with TBS-T for 3 times and incubated with primary antibody (1 : 1000 dilution): anti-insulin and proinsulin (ab14042, Abcam), AT1 (sc-57036, Santa Cruz), and VEGF-A (19003-1-AP, Proteintech) at 4°C overnight. Then membrane was incubated with indicated secondary antibody (1 : 30000) for 1 h. Signal was detected by chemiluminescence using the ECL detection system. Quantification of bands was performed using Image J software.

### 2.9. Immunohistochemistry

Collected islets or pancreas (islets in situ) was incubated with 200 uL rat plasma, coagulated after adding 15 uL thrombin (H32020892). The islet/pancreas specimens were soaked in 4% formaldehyde, after embedding in paraffin; they were sliced into 5 um thick sections. Briefly, after heat-induced antigen retrieval at 95°C for 30 min, the slides were dipped in 0.3% H_2_O_2_ for 10 min to quench the endogenous peroxidase and then incubated in 1% BSA/PBS for 10 min, followed by overnight incubation with indicated primary antibodies at 4°C. The primary antibodies were as follows: anti-insulin and proinsulin (Abcam, ab8304, 1 : 500), anti-CD31 (NB100-64796, Novus Biologicals, 1 : 200), and anti-VEGF-A (19003-1-AP, Proteintech, 1 : 500). Afterward, the slides were incubated at room temperature for 15 min with appropriate horse radish peroxidase- (HRP-) conjugated secondary antibodies. The sections were colourated with DAB and then observed by light microscopy.

### 2.10. Statistical Analysis

Results were expressed as mean ± SD. Comparisons between two groups were assessed by the *t*-test (paired or nonpaired). Statistical significance among three groups was checked by ANOVA, and difference between any two groups was determined by Newman-Keuls test (*q* test) unless the data were not normally distributed, in which case Kruskal-Wallis test (*H* test) was used. All tests were performed by SPSS 18.0 system. *P* value less than 0.05 was considered statistically significant.

## 3. Results

### 3.1. Isolated Islets with Different Sizes Have Distinct Yield and Histological Features

**Figure 1:**
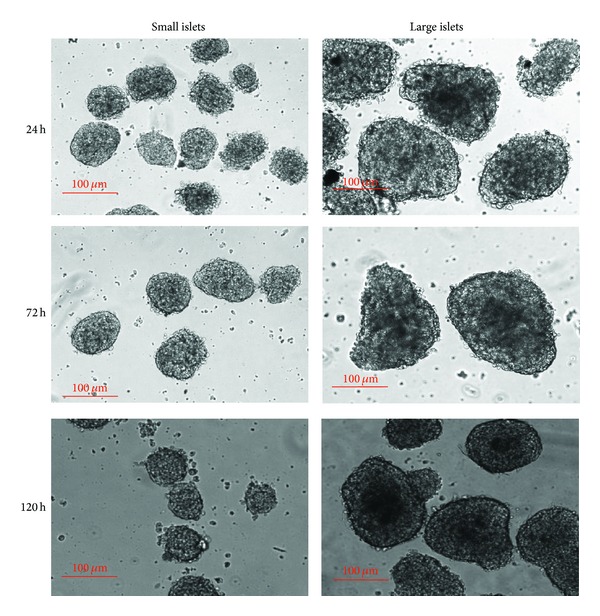
Typical morphology of small and large islets incubated in vitro with indicated time. Note the expanded central reduction of light transmittance within the large ones.

**Figure 2:**
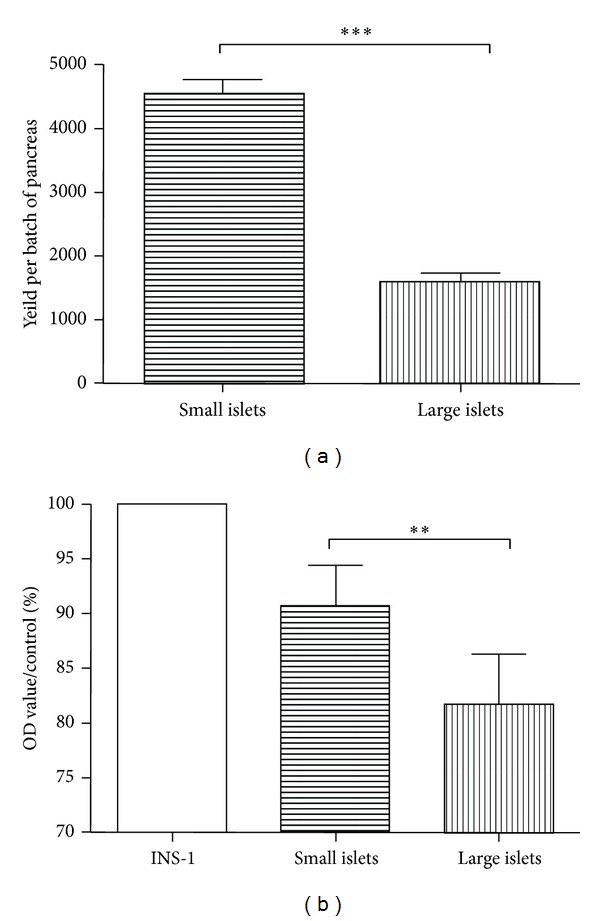
Distinct yield and islet cell viability between large and small islets freshly isolated. (a) Small islets are as twice to three times the amount of large ones isolated from each single batch of pancreas. (b) Islet cell viability analyzed by CKK-8 kit. Islet cells from freshly isolated small grafts are as much as 11% more viable than those from small ones (*P* = 0.0040).

After routine isolation, the size and numbers of each islets can be readily determined and recorded under light microscopy during separation of small islets (with diameters <125 *μ*m) from large ones (with diameters >150 *μ*m). After manual separation of small islets from the large ones, distinct characteristics were noted between these two groups (typical morphology of small and large islets freshly isolated is shown in Figure 1). Consistent with the past study, we observed a significantly greater total amount of small islets from pancreas of each healthy rat. Although the small islets only accounted for 27% of the total IEQs [15], they were about twice to three times the amount of large ones (shown in Figure 2(a)). These indicate the great potential of the small islet grafts in PIT since the small ones have not been paid as much attention as their worth.

### 3.2. Small Islets Freshly Isolated and Cultured In Vitro Survive Better Than Large Ones

Immediately after manual separation, isolated islets were cultured under the same condition. With a 24-hour incubation in vitro, islets were observed under light microscope. Not surprisingly, large and intact islets, rather than small ones, underwent typical core cell death implicated by a reduction in light transmittance morphologically. After a 72-hour incubation, this pattern of cell death was deteriorated and the dark core area expanded in large islets. The typical pattern of cell death was less apparent in small islets until 120 hours later, when the large ones had failed 50% of the total area (see Figure 1).

Further quantified analysis of cell viability by CKK-8 kit revealed that small islet grafts demonstrated significantly increased viability (shown in Figure 2(b)). Consistent with the morphological implication, freshly isolated islets embraced more viable cells compared with large ones (*P* = 0.0040). The difference in viability, of both newly isolated and incubated with prolonged time, further suggests the superiority of small islets in PIT.

### 3.3. Small Islets Embrace More Insulin-Producing *β*-Cells and Function More Vigorously under Glucose Load

**Figure 3:**
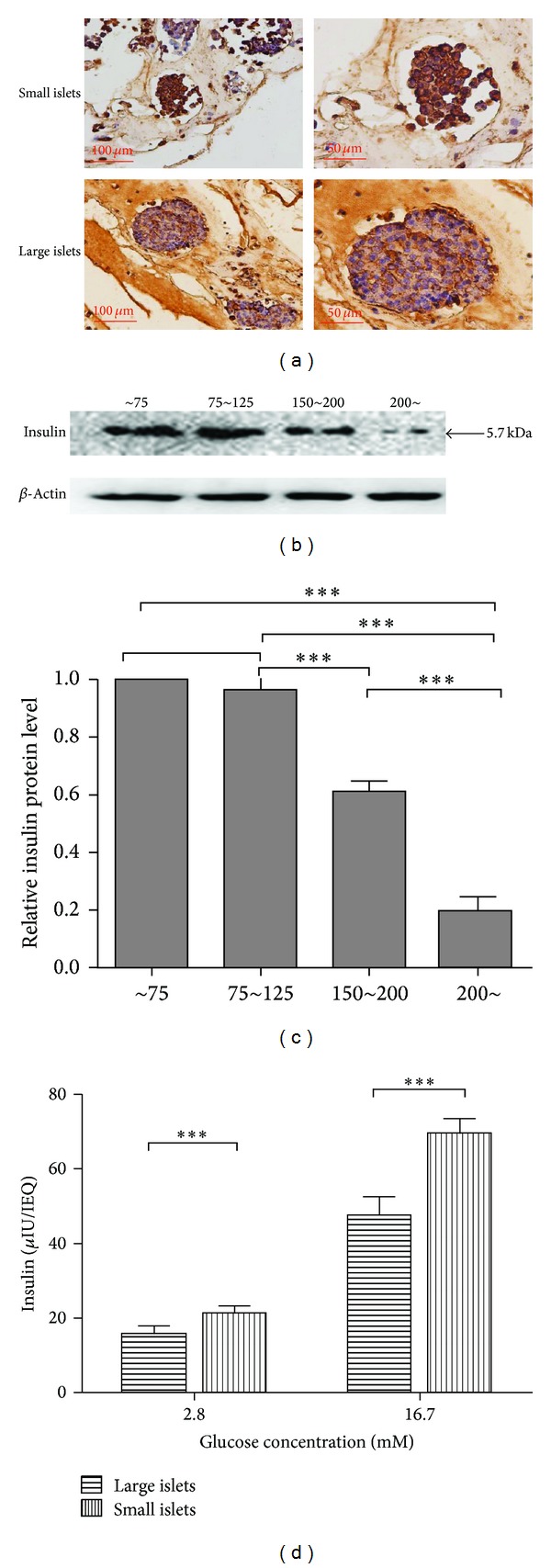
Small islets embrace more insulin-producing *β*-cells and function more vigorously. (a) Insulin immunostaining (brown) of islets in situ in the sectioned pancreas. Small islets showed an apparent more distinct and dense insulin staining compared with the large ones. (b) Immunoblotting of insulin in isolated islets of indicated size. (c) Quantification of insulin immunoblotting above. Small islets have more insulin reservation than large ones normalized to total protein content (*P* < 0.0001). (d) In vitro glucose stimulated insulin secretion assay of isolated islets. Small islets represent more potent insulin-secretion ability both in basal state (2.8 mM glucose) and after 16.7 mM glucose challenge (*P* = 0.0007 and *P* < 0.0001, resp.).

Immunohistochemistry was performed on sectioned pancreas to determine the composition and distribution of insulin-producing cells in small and large islets (shown in Figure 3(a)). Both large and small islets in situ showed a typical distribution of insulin-positive cells. It could be obviously observed in situ that small islets showed a more distinct and intensive insulin staining (brown) compared with the large ones, which is in accordance with the previous study in human. In isolated islets, insulin content was again analyzed by western blotting according to islet size. Our data showed that small islets, normalized to total protein content, have more insulin reservation than large ones (*P* < 0.0001, Figures 3(b) and 3(c)).

To further assess the insulin secretory function in vitro, we determined insulin levels under both basal condition (2.8 mM glucose) and high glucose challenge (16.7 mM glucose). The small islets, once again, demonstrated significantly stronger insulin secretory capabilities than the large ones when normalized to IEQ both basally (15.74 ± 2.05 versus 21.26 ± 1.91 *μ*IU/IEQ, *P* = 0.0007) and after glucose challenge (47.53 ± 4.965 versus 69.42 ± 3.985 *μ*IU/IEQ, *P* < 0.0001) (see Figure 3(c)).

### 3.4. Small Islets Transplantation Is Superior to Large Ones in STZ Induced Diabetic Rat Models

**Figure 4:**
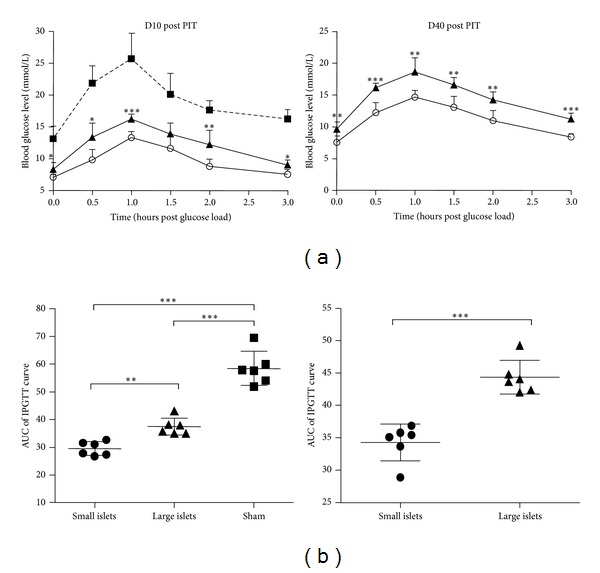
IPGTT of rat recipients after PIT at indicated time points. (a) IPGTT of rat recipients after PIT at d10 and d40, respectively. At the 10th day after PIT, the glucose levels of indicated time after intraperitoneal glucose challenge were significantly higher in large islets PIT group than in small islets PIT group (*P* = 0.0335, 0.0106, 0.0002, 0.0507, 0.0078, and 0.0103 for 0 h, 0.5 h, 1 h, 1.5 h, 2 h, and 3 h, resp.). At the 40th day, the small islets recipient group demonstrated a better glucose tolerance (*P* = 0.0074, 0.0002, 0.0028, 0.002, and 0.0026 and *P* < 0.0001 at 0 h, 0.5 h, 1 h, 1.5 h, 2 h, and 3 h, resp., during IPGTT). (b) Comparison of AUCs calculated from IPGTT curves above also indicated both better short-term effectivity and longevity of small islets transplantation (for both *P* < 0.0001).

We use STZ induced diabetic rats as recipients. Most rats developed diabetes after one administration of 85 mg/kg STZ. The transplantation operations were performed a week after successful induction of diabetes confirmed by tail vein blood glucose monitor for 3 consecutive days. Short-term transplantation effectivity was evaluated by IPGTT on the 10th day after PIT. As seen in Figure 4(a), compared with sham-operated group, PIT effectively improved the glycolic control in STZ induced diabetic rats (*P* < 0.0001). However, diabetic rats transplanted with small syngenic islets demonstrated an almost euglycemic effect while most of those transplanted with large islets remained hyperglycemic. At the 10th day after PIT, the fasting blood glucose levels as well as glucose levels of indicated time after intraperitoneal glucose challenge were significantly higher in large islets PIT group than in small islets PIT group (*P* = 0.0335, 0.0106, 0.0002, 0.0507, 0.0078, and 0.0103 for 0 h, 0.5 h, 1 h, 1.5 h, 2 h, and 3 h, resp.). With the 40-day follow-up, again the small islets recipient group demonstrated better long-term outcomes regarding the better glucose tolerance (*P* = 0.0074, 0.0002, 0.0028, 0.002, and 0.0026 and *P* < 0.0001 at 0 h, 0.5 h, 1 h, 1.5 h, 2 h, and 3 h, resp., during IPGTT) (shown in Figure 4(a)).

The quantified areas under curve (AUC) of blood sugar also indicated both better short-term effectivity and long-term outcome after small islets transplantation therapy (for both *P* < 0.0001, Figure 4(b)) indicating a more vigorous function and better survival of the small islet grafts.

### 3.5. Small Islets with Less Intrinsic Ang II-AT1 Tension Are Nourished by Adequate Microcirculation

**Figure 5:**
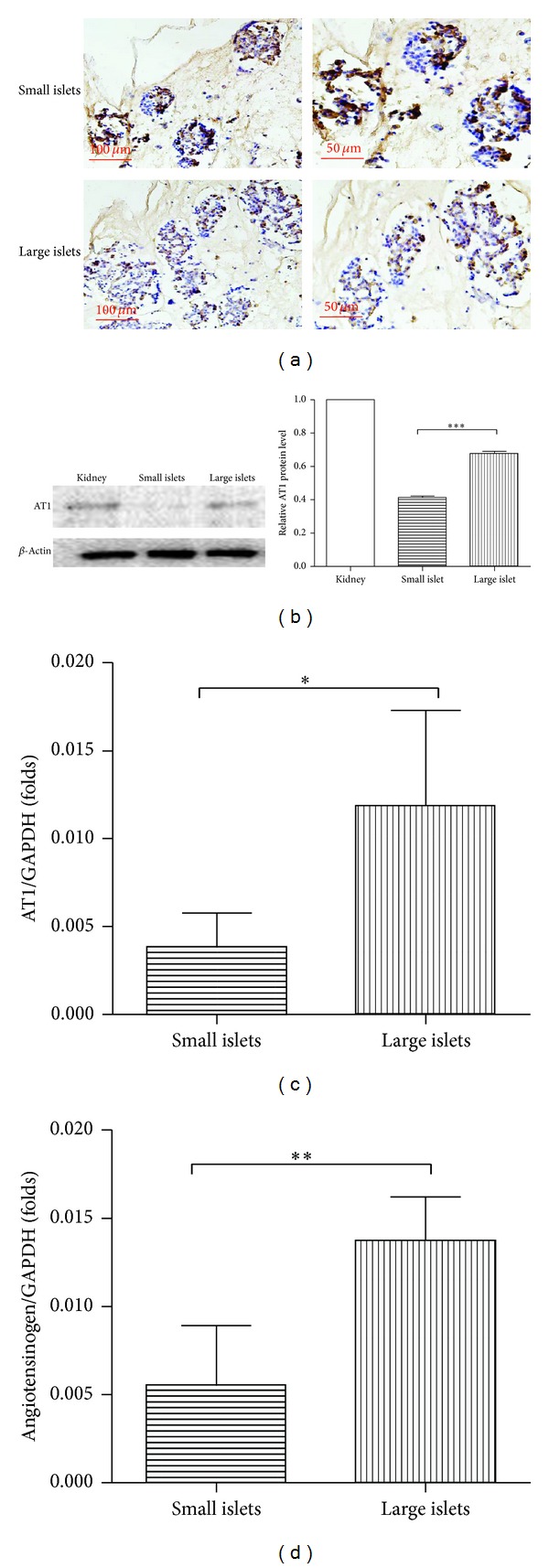
Small islets with less intrinsic Ang II-AT1 tension are nourished by adequate microcirculation. (a) Immunostains of CD31 (brown) in islets in situ from the sectioned pancreas. Small islets are embedded in a more adequate microcirculation reflected by a darker vascular endothelial marker staining. Quantification of local AT1 receptor expression by western blot (b) and qRT-PCR. (c) AT1 receptor is significantly less intensively expressed in small islets at both mRNA (*P* = 0.0321) and protein (*P* = 0.0007) levels. (Protein from the kidney tissue was arranged as positive control.) (d) The qRT-PCR analysis of angiotensinogen. Small islets again expressed less angiotensinogen verified at mRNA levels (*P* = 0.0079).

As previously reported [25], pancreatic islets have an intrinsic expression of angiotensinogen-Ang II-AT1 receptor system. Here we verified the local expression of both angiotensinogenand AT1 gene by qRT-PCR. Angiotensinogen is the precursor of Ang II; the latter has a potent impact on local microcirculation both natively and in the transplanted site [25]. In our study, we first revealed a significantly less amount (about one-third) of angiotensinogen mRNA levels in small islets (*P* = 0.0079, Figure 5(a)). Further the dominant type of Ang II* receptor *AT1 expressed intrinsically in islets was also determined. As shown in Figures 5(b) and 5(c), small islets expressed less* AT1* at both mRNA (*P* = 0.0321) and protein (*P* = 0.0007) levels. This might render them less susceptible to either local or systemic excessive Ang II tension. In our study, this milder local angiotensinogen-Ang II-AT1 expression corresponded to the enriched microcirculation marked by CD31 expression. Our data suggest novel signal mechanisms involved in the distinct groups of islets and their difference as grafts in PIT outcomes.

### 3.6. Enriched Local VEGF-A Expression in Small Islet Grafts Coordinates with Better Transplantation Outcomes

**Figure 6:**
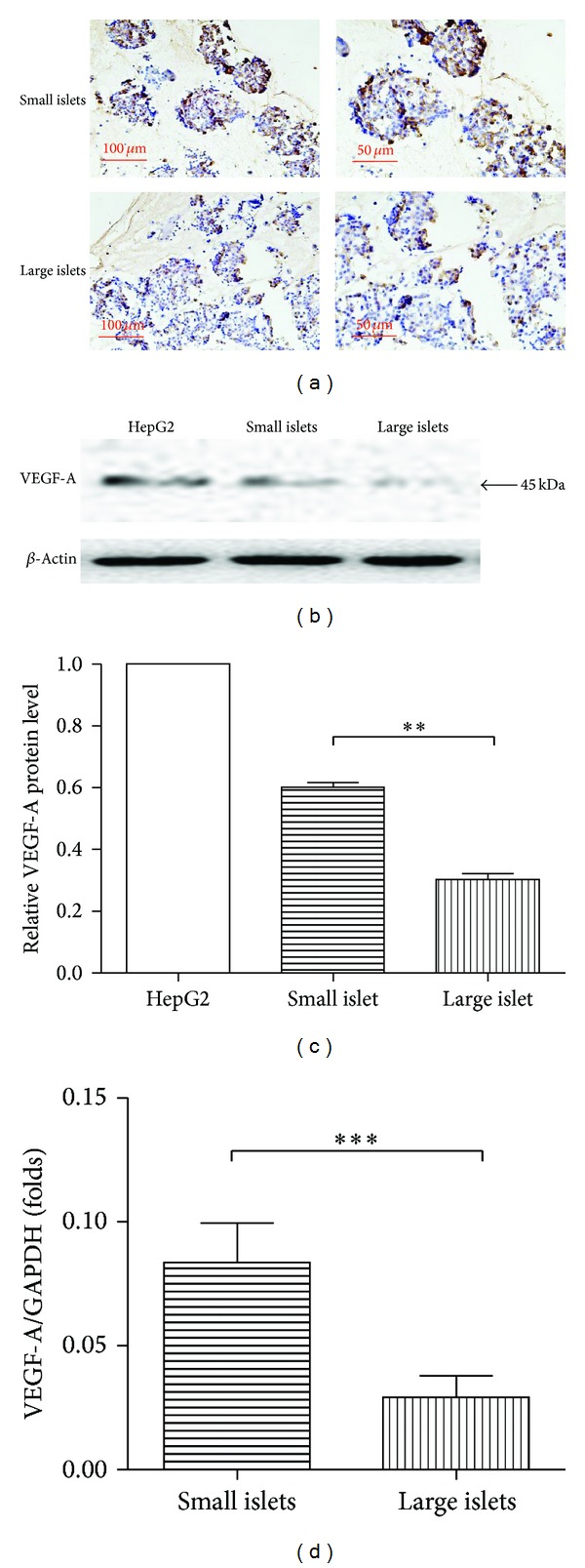
Small islets expressed more abundant VEGF-A. Small islets are apparently enriched in intrinsical VEGF-A expression verified by immunostaining of VEGF-A (brown) in situ and western blot (b and c, *P* = 0.0010) as well as qRT-PCR (d) (*P* = 0.0002) of isolated islets.

The adequate and timely revascularization is critical for islet grafts survival and function; thus factors affecting islet grafts revascularization were intensively explored. VEGF-A, as proved by recent studies, shows a potent and dominant role in promoting vasculogenesis and angiogenesis and improving islet transplantation outcomes [26, 27]. In this study, the difference of local* VEGF-A* expression was explored intensively, both in situ and in isolated islets. As illustrated by in situ immunohistochemical staining (Figure 6(a)), the small islets embraced a stronger expression of VEGF-A (brown staining). The mRNA and protein levels were further explored in isolated islets. In consistence with immunohistochemistry result, VEGF-A was much more abundantly expressed in small islets than large ones confirmed by qRT-PCR (*P* = 0.0010, Figure 6(b)) and immunological blotting (*P* = 0.0002, Figure 6(c); protein from HepG2 cell line was arranged as positive control). The enriched local VEGF-A in small islet grafts coordinated with better short-term transplantation effect and long-term outcomes as shown in Figure 4.

## 4. Discussion

Since the emergence of PIT as a promising cure therapy of diabetes, efforts aimed at making PIT a more competitive alternative to insulin injections have focused on improving the longevity and functionality of islet grafts [10]. Meanwhile, barriers to the use of islet transplantation as a practical treatment for diabetes also include the limited number of available donor grafts [4]. All of these make the careful selection of ideal grafts even more important in the success of the therapy. It is evident in recent years that the small islets are superior in in vitro function and in transplantation outcomes. The most accessible explanation is the physical barrier to diffusion. However, this explanation was rebutted since the reduction of diffusion barriers in isolated rat islets did not improve insulin secretion or transplantation outcome [16].

In the recent years the potential impacts of islet microcirculation, especially islet microvascular endothelium, on *β*-cell fate and function were stressed [20, 28, 29]. The efficient supply of oxygen and nutrients as well as the transport of insulin greatly relies on the unique microvasculature. The microvascular endothelial cells also interface with *β*-cell via localized secretory signals such as vasoactive or angiogenic substances, cytokines, and growth factors, which promote *β*-cell proliferation and affect adult *β*-cell function [30, 31]. In contrast to the physical conditions, in PIT, isolated islets are deprived of their native vascular network [20, 22]. Thus apart from the immunologic attack, the survival and function of islet grafts depend crucially on the timely and adequate process of revascularization [22]. It is reported that, despite the administration of a large quantity of islets mass, more than 70% of the grafts fail to survive within the recipients [1]. Delayed or insufficient revascularization can deprive islets of oxygen and nutrients, contributing dominantly to the loss and failure of the vast majority of grafts shortly after transplantation irrespective of either transplantation sites or islet grafts mass [20–22]. Therefore, it is plausible that differences in microcirculation and revascularization of different islet grafts play an important role in the different transplantation outcomes.

With effort to exploring the differences in microcirculation of different islet grafts, immunohistochemical staining of CD31, which is known as vascular endothelial marker, was performed. Our data first suggest a significant difference in vessel density between small and large islets. To further study the mechanisms underlying, differences in the local angiotensin-generating system and angiotensin II (Ang II) receptor type 1 (AT1) expressions were studied. The intrinsic Ang II-AT1 system in pancreatic islets has been highlighted with an important role in regulating islet blood flow, vascularization, and *β*-cell insulin secretion [21, 25, 32, 33]. Our data revealed for the first time that small islets with less intrinsic Ang II-AT1 tension are nourished by adequate microcirculation, which suggested a novel signal involved in the intrinsic phenotypic divergence as seen between large and small islets.

Among the intensively explored factors involved in islet grafts revascularization, VEGF-A is a well-known subfamily of key angiogenic signal proteins with a dominant role in promoting vasculogenesis and angiogenesis. VEGF-A is intrinsically expressed in the pancreatic islets [34–36]. Expression of VEGF-A in transplanted islets is significantly reduced to 2-3 days after transplantation, coinciding with the delayed and insufficient islet revascularization in diabetic mice [35]. In contrast, enhanced VEGF-A signal improves PIT outcome [26, 27, 32, 34, 37–41]. In our study, enriched local VEGF-A expression in small islet grafts was revealed, coordinating with the better transplantation outcomes in small islets rather than large ones. Altered local expression of VEGF-A provides a logical explanation for distinct graft revascularization and, thus, survival of isolated islet grafts from this functionally unique population.

In our study, we verified a higher density of insulin-producing *β*-cells and correspondingly stronger secretory function in small islets. More importantly, our data revealed for the first time a less tensed Ang II-AT1 signal and more angiotrophic VEGF-A expression intrinsically in small islets compared with large ones. Our data provides novel clues for the molecular mechanisms underlying the transplantation superiority of small islet grafts from the aspects of facilitated microcirculation and revascularization. It also adds bricks to the current knowledge concerning the phenotypically and functionally distinct populations of islets.
